# Bell’s Palsy in Children (BellPIC): protocol for a multicentre, placebo-controlled randomized trial

**DOI:** 10.1186/s12887-016-0702-y

**Published:** 2017-02-13

**Authors:** Franz E. Babl, Mark T. Mackay, Meredith L. Borland, David W. Herd, Amit Kochar, Jason Hort, Arjun Rao, John A. Cheek, Jeremy Furyk, Lisa Barrow, Shane George, Michael Zhang, Kaya Gardiner, Katherine J. Lee, Andrew Davidson, Robert Berkowitz, Frank Sullivan, Emily Porrello, Kim Marie Dalziel, Vicki Anderson, Ed Oakley, Sandy Hopper, Fiona Williams, Catherine Wilson, Amanda Williams, Stuart R Dalziel, for the PREDICT (Paediatric Research In Emergency Departments International Collaborative) research network

**Affiliations:** 1 Department of Emergency Medicine, Royal Children’s Hospital, Flemington Rd, Parkville, VIC 3052 Australia; 2 Murdoch Children’s Research Institute, Parkville, Victoria Australia; 3 Department of Paediatrics, Faculty of Medicine, Dentistry and Health Sciences, University of Melbourne, Melbourne, VIC Australia; 4 Department of Neurology, Royal Children’s Hospital, Parkville, VIC Australia; 5 Princess Margaret Hospital for Children, Perth, Australia; 6 Schools of Paediatric and Child Health and Primary, Aboriginal and Rural Health Care, University of Western Australia, Perth, Western Australia Australia; 7 Lady Cilento Children’s Hospital, Brisbane, Australia; 8 University of Queensland, Brisbane, Australia; 9 Mater Research Institute, Brisbane, Australia; 10 Women’s & Children’s Hospital, Adelaide, Australia; 11 The Children’s Hospital at Westmead, Sydney, Australia; 12 Sydney Children’s Hospital, Randwick, New South Wales, Australia; 13 Monash Medical Centre, Clayton, Victoria, Australia; 14 Townsville Hospital and James Cook University College of Medicine and Dentistry, Townsville, Australia; 15 Sunshine Hospital, St Albans, Victoria Australia; 16 Gold Coast University Hospital, Southport, Queensland Australia; 17 John Hunter Hospital, Newcastle, New South Wales Australia; 18 Department of Anaesthesia, Royal Children’s Hospital, Parkville, Victoria Australia; 19 Department of Otolaryngology, Royal Children’s Hospital, Parkville, Victoria Australia; 20 Department of Family & Community Medicine, North York General Hospital, Dalla Lana School of Public Health, University of Toronto, Toronto, ON Canada; 21 Pharmacy Department, Royal Children’s Hospital, Parkville, Victoria Australia; 22 Centre for Health Policy Melbourne School of Global and Population Health, The University of Melbourne, Carlton, Victoria Australia; 23 Psychological Sciences & Paediatrics, University of Melbourne, The Royal Children’s Hospital, Melbourne, VIC Australia; 24 Starship Hospital, Auckland, New Zealand; 25 Liggins Institute, University of Auckland, Auckland, New Zealand

**Keywords:** Bell’s palsy, Facial nerve palsy, Prednisolone, Randomised controlled trial, Child, House Brackmann scale

## Abstract

**Background:**

Bell’s palsy or acute idiopathic lower motor neurone facial paralysis is characterized by sudden onset paralysis or weakness of the muscles to one side of the face controlled by the facial nerve. While there is high level evidence in adults demonstrating an improvement in the rate of complete recovery of facial nerve function when treated with steroids compared with placebo, similar high level studies on the use of steroids in Bell’s palsy in children are not available. The aim of this study is to assess the utility of steroids in Bell’s palsy in children in a randomised placebo-controlled trial.

**Methods/Design:**

We are conducting a randomised, triple-blinded, placebo controlled trial of the use of prednisolone to improve recovery from Bell’s palsy at 1 month. Study sites are 10 hospitals within the Australian and New Zealand PREDICT (Paediatric Research in Emergency Departments International Collaborative) research network. 540 participants will be enrolled. To be eligible patients need to be aged 6 months to < 18 years and present within 72 hours of onset of clinician diagnosed Bell’s palsy to one of the participating hospital emergency departments. Patients will be excluded in case of current use of or contraindications to steroids or if there is an alternative diagnosis. Participants will receive either prednisolone 1 mg/kg/day to a maximum of 50 mg/day or taste matched placebo for 10 days. The primary outcome is complete recovery by House-Brackmann scale at 1 month. Secondary outcomes include assessment of recovery using the Sunnybrook scale, the emotional and functional wellbeing of the participants using the Pediatric Quality of Life Inventory and Child Health Utility 9D Scale, pain using Faces Pain Scale Revised or visual analogue scales, synkinesis using a synkinesis assessment questionnaire and health utilisation costs at 1, 3 and 6 months. Participants will be tracked to 12 months if not recovered earlier. Data analysis will be by intention to treat with primary outcome presented as differences in proportions and an odds ratio adjusted for site and age.

**Discussion:**

This large multicenter randomised trial will allow the definitive assessment of the efficacy of prednisolone compared with placebo in the treatment of Bell’s palsy in children.

**Trial registration:**

The study is registered with the Australian New Zealand Clinical Trials Registry ACTRN12615000563561 (1 June 2015).

## Background

Bell’s palsy, also known as acute idiopathic lower motor neurone facial paralysis, is characterized by sudden onset paralysis or weakness of the muscles to one side of the face controlled by the facial nerve. Bell’s palsy is an idiopathic, presumed immune mediated phenomenon, possibly with an infection as the inciting event [1–3]. In addition to the muscles of facial expression, the affected seventh cranial nerve in Bell’s palsy also supplies the taste buds to the anterior two thirds of the tongue, muscles associated with hearing, and the salivary and lacrimal glands. The early effect of Bell’s palsy is an inability to fully close the mouth and eye on the affected side of the face, causing difficulties in eating and speaking, corneal drying and erosion. Later symptoms can include pain around the ear sometimes extending to the back of head/neck, altered taste, synkinesis (involuntary facial movements), facial spasm, facial contracture, dysfunctional lacrimation (corneal drying and/or crocodile tears) and noise intolerance [2]. The resulting impairment of oral competence, verbal communication and social interaction, can contribute to significant emotional distress during Bell’s palsy [4–7].

Data from the United Kingdom reported an incidence of Bell’s palsy of more than 6 per 100,000 person-years in children under 14 years and more than 20 per 100,000 person-years in people aged 15–29 years [8], although the true incidence may be higher [9]. In an emergency department based study of stroke mimics in children [10] Bell’s palsy was the third most frequent diagnosis in children with sudden onset neurological dysfunction. In adults, up to 30 % of untreated patients fail to make a complete recovery [11]. While in children up to 100 % recover from Bell’s palsy within 12 months, it often leaves them with a prolonged period of functional impairment, disfigurement and emotional distress [4, 12–16].

The main agents used for the treatment of Bell’s palsy are steroids. The anti-inflammatory effect of steroids (such as prednisolone) is assumed to minimize facial nerve swelling, compression and damage, therefore reducing the length of time to and increasing the likelihood of recovery [2, 17]. Two recent, large, blinded randomised controlled trial (RCTs) of steroids in adults [18, 19] (combined *n* = 1309) reported statistically and clinically significant improvements in the proportion who reached complete recovery when Bell’s palsy was treated with steroids compared with placebo. The participants in both studies were randomised to receive prednisolone and acyclovir, prednisolone and placebo, acyclovir and placebo or placebo and placebo. One of the studies, by Engstrom et al. (839 patients), showed shorter times to recovery for patients who received prednisolone compared with those who did not (combining those who did and did not receive acyclovir, hazard ratio 1.4 95 % confidence interval [CI] 1.18 to 1.64; *p* < 0.0001) [19]. The other study (Sullivan et al., 496 patients) found that more patients had complete recovery by 3 months in the group who received prednisolone (83 %) compared with placebo (63 %) (again combining participants who did and did not receive acyclovir, difference 19.4 %; 95 % CI: 11.7 to 27.1 %); number needed to treat to achieve one additional complete recovery was 6 (95 % CI: 4 to 9) [18]. A recent (2010) Cochrane review (8 trials, *n* = 1569) on treatment for Bell’s palsy [17], including the two RCTs above, found that corticosteroid treated patients had a lower risk of incomplete recovery than placebo treated patients (relative risk [RR] 0.71; 95 % CI 0.61 to 0.83) and that those treated with corticosteroids also had reduced risk of synkinesis during follow up (RR 0.6; 95 % CI 0.44 to 0.81). Subsequently, in 2012 the *American Academy of Neurology* released a guideline update on the use of steroids and antivirals to treat Bell’s palsy [20]. It concluded that steroids have proven efficacy in treating Bell’s palsy in adults, and that no further research is required. Similarly, in 2013 the *American Academy of Otolaryngology-Head and Neck Surgery Foundation* published a clinical practice guideline recommending the use of oral steroids within 72 hours in patients with Bell’s palsy 16 years and older [21].

Despite the conclusive evidence of benefit from steroids in adults with Bell’s palsy, two systematic review of the use of steroids to treat Bell’s palsy in children found no placebo controlled trials [13, 22]. There has only been one small randomised trial of steroid use in children with Bell’s palsy. Unüvar et al. [12] randomised 42 children (mean age 53 months) to receive 1 mg/kg/day of methylprednisolone or no treatment (no placebo). The study found that all children fully recovered within 12 months regardless of treatment. However, at 4 and 6 months after treatment, more children in the methylprednisolone group had recovered compared with the no treatment group (86 vs 72 % at 4 months and 100 vs 85 % at 6 months). These findings suggest that although children eventually recover, steroids hasten the time to recovery. The lack of a placebo arm, small patient numbers, and inclusion of only children with severe signs and symptoms at presentation limit the generalisability of these results. Two systematic reviews of the use of steroids to treat Bell’s palsy in children found no placebo controlled trials and either state that there was insufficient evidence to make a treatment recommendation [13] or provide no positive evidence for the beneficial effects of corticosteroids and do not recommend the routine use of steroids in children [22]. The results from adult studies of steroids to treat Bell’s palsy are difficult to extrapolate to children as medical conditions often manifest differently and have different responses to treatment in adults and children. For example, there are documented differences from adults in the pathophysiology [23] and natural history of other paediatric demyelinating conditions: (i) acute disseminated encephalomyelitis, which is typically monophasic, has different presenting features and involves different brain structures than similar diseases in adults (such as multiple sclerosis) [24, 25]; (ii) acute myelitis can have a good prognosis in children but usually has a poor prognosis in adults [26]. Similarly, it is unclear whether the pathophysiology in children is different from adults.

Both systematic reviews of the use of steroids to treat Bell’s palsy in children [13, 22] call for a definitive trial to be conducted. The *American Academy of Neurology* similarly called for RCTs to determine if Bell’s palsy in children should be treated with steroids [20]. The *American Academy of Otolaryngology-Head and Neck Surgery Foundation* clinical practice guideline states that corticosteroids may be considered in pediatric patients with a large role for care giver involvement in the decision making process [21]. A consequence of the lack of evidence and firm treatment recommendations is variable use of steroid in children reported from a number of centers [4, 13, 14, 16].

Based on the lack of placebo-controlled evidence for or against steroid use in Bell’s palsy in children and the resulting variability in care, and the high level evidence of the efficacy of prednisolone in adults we designed a study with the primary aim to determine, in children aged between 6 months and less than 18 years presenting to the emergency department (ED) with symptoms of Bell’s palsy (unilateral, peripheral facial nerve palsy), whether treatment with oral prednisolone (at ~1 mg/kg daily for 10 days) increases the proportion of children who have complete recovery at 1 month (defined as grade 1 on the House-Brackmann (HB) scale [18, 27]) compared with children receiving placebo. This study will also provide a unique insight into Bell’s palsy related questions including the time to complete recovery; the emotional and functional impact of Bell’s palsy; the effect of initial severity and time to treatment on subsequent recovery; and the nature of pain, motor synkinesis and autonomic dysfunction in children with Bell’s palsy.

## Methods

### Study design

This is a phase III, triple-blinded, randomised, placebo-controlled superiority trial of prednisolone for treatment of Bell’s palsy. Participants will be randomised in a 1:1 ratio to receive ~ 1 mg/kg/day of prednisolone, up to a maximum of 50 mg, or matched placebo for 10 days. The study will include a total of 540 children (approximately 270 in each treatment group). This sample size allows for 10 % loss to follow-up at 1 month. The study will be conducted at sites of the Paediatric Research in Emergency Departments International Collaborative (PREDICT) research network in Australia and New Zealand [28]. The central study site is the Murdoch Childrens Research Institute in Melbourne (MCRI), Australia and the central study pharmacy is at the co-located Royal Children’s Hospital (RCH), Melbourne, Australia.

### Outcomes

#### Primary outcome

The primary outcome is complete recovery at 1 month post randomisation, where recovery is defined as a House Brackmann score of 1 [18, 27]. Recovery will be assessed face-to-face by a site specialist clinician who may be a pediatric neurologist, ear-nose-throat (ENT) specialist, pediatrician or ED physician. Where the participant is unable to attend the study site, it may be completed via videoconference using an online tool.

There are several different tools used in studies to describe the degree of facial dysfunction of Bell’s palsy, including the House Brackmann (HB) [12, 18, 19, 29–31], Sunnybrook [19, 32], Facial Paralysis Recovery Index/Profile [29, 33], and Yanagihara grading systems [34, 35]. The two largest adult RCTs used Sunnybrook scale and HB scale, or the HB scale alone [18, 19], and the only RCT of steroids for treating Bell’s palsy in children from Unüvar et al. [12] used the HB scale to assess recovery. In line with these studies, the primary tool to determine outcome in this study is the HB scale as used slightly modified by Sullivan et al. [36].

Using the HB scale, the two largest adult studies defined recovery as reaching a HB grade of 1 (HB 1) [18, 19]; others, including Unüvar [12], used HB grade 1 or 2. Sullivan et al. [18] initially set ‘complete recovery’ at HB grade 1 or 2, however, very early in the study the investigators found that participants only looked and felt fully recovered once HB grade 1 was obtained and hence changed their definition to HB grade 1. Our study accordingly defines full recovery as having achieved HB grade 1.

There are currently limited data regarding how long it takes children to recover from Bell’s palsy but existing data suggest a more rapid recovery in children than in adults [4, 12, 14, 37]. Unüvar’s study of 42 children [12] found that 72 % of children had complete recovery at four months, 85 % at six months, and 100 % recovery at 12 months without steroids. In two paediatric observational studies [4, 14], the mean time to recovery was five weeks (range 1–21 weeks) and 59 % had recovered at 1 month without steroids. Therefore the primary outcome in the current study was chosen as complete recovery at 1 month with recovery at 3 and 6 months as secondary outcomes.

#### Secondary outcomes

The secondary outcomes are:

1. Complete recovery at 1 month using the Sunnybrook scale [32, 38]
2. Complete recovery at 3 months using the Sunnybrook scale
3. Complete recovery at 3 months using the HB scale
4. Complete recovery at 6 months using the Sunnybrook scale
5. Complete recovery at 6 months using the HB scale
6. Complete recovery at 1, 3 and 6 months using the HB scale according to age, time to treatment and initial severity
7. Parent/guardian and participant (where aged ≥8 years) perception of facial nerve recovery at 1, 3 and 6 months using a lay translation of the HB scale
8. Emotional and functional wellbeing of the participant assessed by the parent/guardian and participant using the Pediatric Quality of Life Inventory (PedsQL) [39] and Child Health Utility 9D (CHU9D)scales [40–43] and sections of the Harter scale at 1,3, and 6 months [44].
9. Pain at 1, 3 and 6 months assessed using child assigned visual analogue scale or Faces Pain Scale Revised (for participants aged 5 and older) and using a parent assigned visual analogue scale (VAS) [45–47]. Both pain scales are numbered 0–10.
10. Prevalence of synkinesis or autonomic dysfunction at 1, 3 and 6 months using the Sunnybrook scale augmented by a synkinesis assessment questionnaire (SAQ) [48]
11. Health utilization costs assessed via CHUD9 and via capture of information from the parent/guardian/participant related to in-patient, out-patient, or ED visits and to any other health facilities including general practitioner attendance for treatment or investigation during the 6 months following randomization.
12. For patients not recovered at 6 months, recovery at 12 months as perceived by parents/guardian and participant (where aged ≥8 years) using a lay translation of the HB scale.

### Study population

Eligible patients must be between 6 months and less than 18 years of age with ED clinician diagnosed Bell’s palsy with onset less than 72 hours before randomisation. ED clinicians will follow local guidelines in the diagnosis of Bell’s palsy. Participants must fulfil all of the inclusion criteria and none of the exclusion criteria (Table 1).

**Table 1:** Inclusion and Exclusion Criteria

Inclusion criteria	
1. Be aged from 6 months to less than 18 years of age.	
2. Weight ≥5 kg.	
3. Be diagnosed with Bell’s palsy by their treating doctor.	
4. Have acute onset of symptoms of Bell’s palsy for less than 72 hours prior to randomisation.	
5. Have written informed consent provided by the parent(s) or legal guardian. The participant, where he/she is deemed competent to provide consent, may also provide written consent.	
Exclusion criteria	
1. Likely to be unable to complete the 1 month study assessment of Bell’s palsy symptoms. Where the participant is unable to attend the study site, the assessment can be completed via videoconferencing using skype software or other online tools.	
2. Previously randomised into the study.	
3. Contraindication to prednisolone, including: active or latent tuberculosis, systemic fungal infection, known hypersensitivity to prednisolone or any of the excipients in the liquid, diminished cardiac function, diabetes mellitus, peptic ulcer or chronic renal failure, multiple sclerosis, recent active herpes zoster or chickenpox.	
4. Use of any systemic or inhaled steroid within 2 weeks prior to the onset of symptoms.	
5. Current or past oncological diagnosis.	
6. Pregnancy.	
7. Breast feeding.	
8. Currently receiving concomitant medications in which prednisolone is contraindicated.	
9. Immunisation with a live vaccine within the previous 1 month.	
10. Requirement for a live vaccine within 6 weeks of the first dose of study drug.	
11. Signs of upper motor neurone VII nerve palsy (weakness of the lower half of the face only).	
12. Diagnosis by a medical doctor of acute otitis media concurrently or within 1 week prior to the onset of Bell’s palsy symptoms.	
13. Evidence of vesicles on the ear drum suggestive of herpes zoster related Ramsay Hunt syndrome.	
14. Known facial trauma within 1 week prior to the onset of symptoms that in the view of the clinician may have caused or contributed to facial palsy.	
15. Any other condition at risk of being influenced by the study treatment or that might affect completion of the study.	
16. Any concern regarding parent/guardian/participant ability to comply with the study protocol.	
17. Prior episode of Bell’s palsy.	

### Randomisation

Participants will be randomized in a 1:1 ratio to one of two treatment arms: intervention (prednisolone) or matched placebo. An independent statistician has generated a randomization schedule using variable block sizes, stratified by site. This schedule will be used by the central study pharmacist at RCH to undertake the blinding, labelling and distribution of the study drugs to participating sites by putting together study packs of the required drug labelled with sequential study numbers for each site and dosing instructions. Randomization will be undertaken at the site by ED and research staff by selecting the lowest numbered study pack (next pack system) from the study drug store in ED.

### Interventions

There is currently little evidence to assist in determining the best prednisolone dose in children. Adult studies of Bell’s palsy have used various steroids and doses [17–19, 29, 49]. The two large adult RCT used 50 mg prednisolone daily for 10 days [18] or 60 mg prednisolone daily for 5 days then weaning over 5 days [19]. The only paediatric RCT of steroid use for Bell’s palsy used methylprednisolone 1 mg/kg/day (equivalent to 1.25 mg/kg/day prednisolone) for 10 days, then weaned over 3–5 days [12]. Observational data from the central study site [4], and unpublished survey data from the PREDICT network indicate that prednisolone is the most frequently used steroid for Bell’s palsy in children, usually at a dose of 1 mg/kg/day for a variable number of days. Prednisolone is also the most frequently used oral corticosteroid in children in Australia and New Zealand for various other inflammatory conditions, with routine doses of 1–2 mg/kg/day.

Noting the adult dose used by Sullivan et al. [18] (50 mg for 10 days without taper); having sought advice from endocrinologists and rheumatologists; in consideration of the current local guidelines [50] and with consideration to ease of preparation of study drugs, a dose of ~1 mg/kg daily for 10 days without taper was chosen for the current study. For the 10-day study treatment period, participants will therefore be assigned to receive either ~1 mg/kg/day of prednisolone (based on weight categories) up to a maximum of 50 mg/day or to receive matching placebo.

Adverse events from this dose and short duration are expected to be limited to minor behavioral changes and possible weight gain. Drug related adverse events will be recorded. Risks should be reduced by adherence to inclusion and exclusion criteria listed above.

Randomization will be blinded so that trial participants, the investigator team (including the facial image assessors), data management staff, data analysis staff and any other clinical staff remain unaware of the study arm to which trial participants have been assigned to. Only the central pharmacist at RCH and the independent statistician who generated the randomisation list will be unblinded.

Prednisolone will be supplied as Redipred® oral liquid. Redipred® contains the active ingredient prednisolone sodium phosphate 6.72 mg/mL (equivalent to prednisolone 5 mg/mL). The look- and taste-matched placebo will be supplied in 30 mL plastic bottles. The Redipred® and matching placebo will be supplied by Aspen Pharmacare Pty Ltd (St Leonards, NSW, Australia).

All participants will receive the study drug (prednisolone or placebo) orally in liquid form. Participants will receive the number of milliliters required per day based on a pre-calculated weight-based table. The study drug will be prescribed as a once daily dose.

The central pharmacist at RCH will blind and label the bottles of prednisolone and placebo and place them into study packs. The central pharmacist at RCH will provide each site’s clinical trial pharmacy with sufficient stock to cover the site’s projected recruitment, co-ordinate ongoing resupply of study medication, ensure in-date supplies are available at each site and oversee drug accountability. Study packs will be kept in each site’s ED in a locked, limited-access facility available for immediate use for participant randomisation.

At the time of randomisation, the senior staff member or medically qualified investigator in the ED will select the lowest numbered study pack available from the study drug store and arrange the first dose of the study drug to be administered in the ED by a clinical nurse. The parent/guardian/participant will be provided with the remaining study drug along with instructions regarding dosing and storage.

#### Adherence/Compliance

Compliance/adherence will be assessed as part of the Day 14 assessment. Families will be instructed to return all study drug bottles at the 1 month study clinic visit when the remaining liquid in the bottles will be measured.

#### Concomitant care

All parent/guardian/participants will be provided with a study alert card for non-study health care professionals providing care during the study.

#### Emergency unblinding

The randomisation code for an individual participant may only be unblinded in emergency situations, where the investigator decides a participant cannot be adequately treated without knowing the identity of their treatment allocation. The randomisation code is held at the central pharmacy at RCH.

### Study procedures and assessments

#### Recruitment and consent

In the event of the presentation of a child with suspected Bell’s palsy ED staff at participating sites will be asked to notify the senior medical doctor on duty who will perform an initial assessment of potential eligibility. If potentially eligible, the senior doctor or a member of the investigator team will approach the participant and their parent/guardian(s) to inform them of the study, determine their interest in participating, confirm eligibility and (where applicable) conduct the written informed consent process.

The relevant clinical teams at each site will receive standardized, study specific education based on centrally developed study education materials. Notification letters about the study will be sent to relevant physicians with consultation or referral roles at study sites.

### Study procedures

An overview and the timing of the study procedures are outlined in detail in Table 2.

**Table 2:** Assessment Schedule BellPIC Study

Procedure	Enrolment & Randomisation Time point 1	Time point 2	Time point 3	Time point 4	Time point 5	Time point 6
	Day 1	Day 14^a^	Month 1^b^	Month 3^b^	Month 6^b^	Month 12^d^
Obtain Informed consent	✓					
Collect demographic and relevant information	✓					
Facial images	✓		✓	✓	✓	
House Brackmann [18, 27] & Sunnybrook [32, 38] scales augmented with SAQ [48]	✓		✓	✓	✓	
Review inclusion & exclusion criteria	✓					
Randomise	✓					
Study drug dispense	✓					
Study drug compliance review		✓	✓			
Adverse events review		✓	✓^e^			
Specialist clinician review			✓^c^			
Emotional and functional impact using PedsQL, CHU-9D and Harter scales [39–44]			✓	✓	✓	
Assessment of pain (VAS/FPS-R) [45–47]			✓	✓	✓	
Participant and parent perception of recovery (lay translation of HB scale)		✓	✓	✓	✓	✓
Collection of information on medical attendances			✓	✓	✓	

*HB* House Brackmann scale, *SAQ* Synkinesis Assessment Questionnaire, *PedsQL* Pediatric Quality of Life inventory, *CHU-9D* Child Health Utility 9D, *VAS* Visual Analogue Scale, *FPS-R* Faces Pain Scale Revised

^a^This assessment will be conducted over the telephone or on-line. A window of ±5 days is allowed

^b^A window of ± 7 days will be allowed to facilitate appointment scheduling for the study assessments attended on-site at 1 month; a window of ±14 days will be allowed to facilitate appointment scheduling or follow up contacts by phone, email or online for study visits or contacts at 3 and 6 months. Once a participant has been assessed to have made a complete recovery (HB score of 1), they will no longer be required to attend further visits to the study clinic and will only be required to complete questionnaires and scales by telephone, email or online at 3 and 6 months

^c^The senior specialist clinician may be a pediatric neurologist; ENT specialist; pediatrician; or ED physician

^d^This assessment will be required only for those deemed to have been not recovered at their 6 month assessment. A window of ±14 days will be allowed to facilitate follow up contacts scheduling

^e^Adverse events which are unresolved at 1 month should be followed till resolution or stabilisation

#### Baseline assessment and randomisation during the ED visit

Following informed consent, eligibility check and randomisation, demographics and relevant clinical information will be collected from the patient including side of the palsy, onset and associated symptoms, relevant past medical history, and medical evaluations/contacts prior to the ED visit.

Study assessments regarding the severity of facial palsy as measured by the HB and Sunnybrook scales and facial imaging (digital video recording and still photographs) will be conducted using standardized instructions based on the study by Sullivan et al. [36]. A camera will be provided to all study sites. The images will be assessed at a later time point by assessors blinded to the study treatment assignment for quality control purposes.

#### Follow up assessment at 14 days

Approximately 14 days after randomization, the parent/guardian/participant will receive a phone call from the study team to assess medication compliance, adverse events and ongoing palsy symptoms (as judged subjectively by the parent/guardian/participant).

#### Follow up assessment at 1 month

One month after randomization, participants will attend a visit at the study site. Where the participant is unable to attend the study site, this may be completed via videoconferencing.

A specialist clinician (a pediatric neurologist; ENT specialist; pediatrician or ED physician to accommodate site specific resources) will review the participant to: exclude an alternative diagnosis for the Bell’s palsy symptoms; determine if Bell’s palsy symptoms are still present; determine HB grade (the primary outcome) and the Sunnybrook scale of the Bell’s palsy; determine the presence of synkinesis or autonomic dysfunction using the SAQ. Facial images (video recording and still photographs) will be recorded. A questionnaire will be completed by the parent/guardian or participant (age dependent) for all participants and will elicit details on adverse events, ongoing palsy symptoms, reports of abnormal hearing, lacrimation and altered taste, date of resolution of facial palsy symptoms (if resolved) as subjectively perceived by parent and any medical contact.

The following scales and tools will be completed by the parent/guardian or participant depending on the age of the participant (varies by scale):

- o PedsQL and CHU-9D to assess the emotional and functional wellbeing (quality of life) of the participant.
- o The Harter scale to assess perceived appearance.
- o FPS-R or VAS to capture pain symptoms
- o A study-specific lay translation of the HB scale

A site research assistant will collect the study drug containers from participants attending the study site for later assessment of residual volumes and return to the site pharmacy.

#### Follow up assessment at 3 and 6 months

A site research assistant will arrange a follow-up assessment for any participants deemed at their previous visit not to be fully recovered (HB score greater than 1). For participants unable to attend the study site, assessment will be conducted remotely as previously described.

Participants deemed to have fully recovered at the 1 month visit will not be required to attend the study site for the remaining study time points; the parent/guardian or participants (age dependent) will instead be asked to complete the questionnaires online or via mail (or via telephone, administered by the research assistant if required). The questionnaires will be the same as those completed by the unrecovered participants but without HB, Sunnybrook, SAQ scoring or facial imaging. Similarly, participants who have fully recovered at the 3 month visit will not be required to attend the study site for the 6 month time point and the parent/guardian/participant will instead be asked to complete the questionnaires online or via mail (or via telephone, administered by the research assistant if required).

Parent/guardian or participant questionnaires, grading scales and facial imaging will be performed similar to the 1 month visit (see Table 1).

#### Follow up assessment at 12 months

For participants not recovered at 6 months, a further contact will be conducted with the parent/guardian or participant with a request to complete a questionnaire on-line or by phone to elicit information on parent perceived recovery of Bell’s palsy. While this data point is outside the RCT period of 6 months, this will allow the determination of the 12 month recovery rate for Bell’s palsy in children which is unknown at this time.

### Statistical methods

#### Sample size estimation

Based on observational data [4, 14] we expect 60 % of children without prednisolone to have complete recovery at 1 month. This is in line with data from the pediatric RCT which reported complete recovery without prednisolone in 72 % at 4 months [12]. A study in adults [18] deemed an improvement of 12 % with prednisolone compared with placebo to be a clinically important difference between treatment groups. We therefore power our study to find an increase in the proportion with complete recovery from 60 % in the placebo group to 72 % in the prednisolone group. This is a conservative estimate of efficacy compared with that found in our observational pilot data [4, 14] which showed an increase in complete recovery at 1 month of 16 % in those treated with prednisolone (75 %) compared with those not receiving prednisolone (59 %). To enable us to identify an increase in recovery from 60 to 72 % or larger with 80 % power requires a study with 244 subjects in each treatment group based on a two-sided test with α = 0.05. Hence we aim to recruit 270 per group (540 in total) to allow for 10 % loss to follow-up at 1 month.

#### Statistical analysis

Data will be analysed on an intention-to-treat basis. For the primary analysis, multiple imputation will be used to handle missing data, imputing all outcomes using a single (joint) imputation model. Imputation will be carried out separately by treatment arm to ensure that any treatment effects are maintained. As a secondary analysis, all analyses will be repeated using a complete case analysis, including all participants with data available for each outcome.

The primary outcome of complete recovery at 1 month post randomization on the HB scale (as determined by the site specialist clinician) will be presented as a proportion in each treatment group, with a comparison between the groups presented as a difference in proportions and as an odds ratio from a logistic regression model adjusted for site and age, with a 95 % CI and *p*-value. In addition, we will present the results as Number-Needed-to-Treat (NNT) and its 95 % CI. As an additional sensitivity analysis, the analysis of recovery at 1 month will be repeated assuming that all participants with missing data who have not previously been recorded as achieving complete recovery 1) have recovered, and 2) have not recovered.

All secondary outcomes will be summarized by treatment group. Binary outcomes will be presented as proportions, with comparisons between the groups presented as a difference in proportions and as odds ratios from logistic regression adjusted for site, with 95 % CIs and *p*-values. Continuous outcomes will be presented as means and standard deviations, with comparisons made using linear regression adjusted for site presented as mean differences, 95 % CIs and *p*-values. Time to recovery will be assessed using a Cox proportional hazards model censoring patients who have not recovered at 6 months, with results presented as a hazard ratio, and its 95 % CI and *p*-value. Participants who have reached complete recovery at 1 or 3 months and are not assessed at the later time points will be assumed to have complete recovery for the duration of the study. For both the primary and secondary outcome, we will repeat the analysis adjusted for age (as a continuous variable), gender, baseline severity of facial nerve dysfunction (severe = HB V and VI, non severe = HB I-IV [18, [36]) and time to treatment (≥24 vs >24–72 hours [18, [36]) as potentially important confounders.

Whether the effectiveness of the intervention in improving the proportion recovered at 1, 3 and 6 months varies according to age, time to treatment and initial severity will be assessed by the addition of an interaction between treatment and the covariate to the logistic regression model.

Face-to-face and video HB scores and the HB vs Sunnybrook scoring will be compared using scatterplots and kappa statistics.

Finally, the effect of prednisolone compared with placebo on health economic markers will be assessed using decision analysis using a comparison of the 6 month treatment cost data and results from the CHU9D administered at 1 month, 3 months and 6 month.

#### Interim analysis and stopping rules

The study will be monitored by an independent Data and Safety Monitoring Board (DSMB). A single interim analysis of the primary outcome will be undertaken and reported to the DSMB after 50 % of participants have completed the 1 month assessment (i.e. primary outcome data available for 244 participants). The Haybittle-Peto rule [51] will be used as a guideline for the DSMB, where the DSMB may recommend the trial be stopped if the level of significance of 0.001 is reached.

### Data management

All study data will be captured on study specific case report forms recorded from the participants medical notes (source data) or parental/participant questionnaires (source data) and will be entered into and stored in a single password protected online REDCap database with data entry conducted at each site. Hard copy participant data at each site will be stored in locked cabinets. REDCap is hosted by MCRI. Information will be stored and retained according to local and government regulations.

### Data monitoring

The Chief Principal Investigator (PI) or designee will conduct site visits in order to review a selection of the database against source data, verify adherence to the protocol and confirm that research governance principles are being upheld. Data management of entered patients will be undertaken by the central data management team and reviewed for completion.

### Safety

Any untoward medical occurrence in a patient enrolled into this study regardless of its causal relationship to study treatment will be recorded. Conditions that are present at screening and do not deteriorate will not be considered adverse events (AEs). The parent/guardian/participant will be asked to report AE when contacted at 14 days and at the 1 month follow up visit. In addition, AEs noted during the visit or noted from any other documentation (e.g. hospital unit record and correspondence) will be documented on the CRF by the investigator team.

The site PI is responsible for recording and reporting all serious adverse events (SAEs) for the period from the first dose until the 1 month visit. Any SAE occurring in a study participant will be reported to the approving Human Research and Ethics Committee (HREC) in accordance with the safety reporting policy of the HREC (this timeframe is usually between 24 and 72 hours). A suspected unexpected serious adverse event will in addition be reported to the national government regulatory body (Australia or New Zealand) within the required timelines.

### Study oversight

Central study coordination will be provided by the MCRI based study team. The trial will be overseen by a trial steering committee (TSC) to monitor and supervise progress of the trial; review at regular intervals relevant information from other sources; and consider the recommendations of the DSMB.

The DSMB has an agreed upon charter and will meet six months after commencing the trial and then every 12 months for the duration of the trial. The DSMB will review data on safety, feasibility and protocol violations by treatment arm but with treatment arms masked. Once primary outcome data are available for 244 participants the DSMB will in addition review the efficacy of the treatment arms using the stopping rule outlined above.

The trial will be coordinated at each site by an on-site Study Management Team consisting of the site PI and a site study coordinator/research assistant. Handling of investigational products will be the responsibility of an on-site pharmacist.

#### Quality control and quality assurance

To standardise the collection of data by local investigator teams, training of the site PI and research assistant will be undertaken by the central coordinating team; the site PI and site research assistant will train the remaining local investigator team in particular the senior ED staff conducting consent and randomisation as well as the specialist clinician(s) reviewing the participant at 1 month.

Equipment for facial images (still photographs and video recordings) will be standardised with the same model of camera supplied to each participating site. Camera and video standard settings instructions along with full usage instructions will be provided to the local investigator teams. The local teams will not assess the facial images for palsy but upload the images to the study database for assessment by 3 central blinded assessors for the purpose of assessing interrater reliability and comparison of HB scoring by face-to-face assessment versus facial images.

### Ethics and dissemination

#### Research ethics approval

The trial has full ethics approval at the central study site from the Royal Children’s Hospital (RCH) Human Research Ethics Committee (HREC reference number RCH HREC/15/RCHM/V4). RCH HREC is also the central site for the National Ethics Application Form. The study has ethics and governance approval at all 10 sites.

#### Consent

Parents and adolescents 12 years and older will be given a parent/guardian/patient information sheet. After discussion with the family, written informed consent will be obtained by the trained clinician and/or researcher in the ED. The study investigators and/or research officer may be involved as clinicians in the clinical care of the patient. Parents/participants will be assured that if they do not wish to participate this will not affect their care.

#### Confidentiality

Confidentiality will be ensured by storing data in a password-protected database for which only the research team will have the password, and paper based record forms will be stored in locked study cupboards and offices. REDcap, the research data base used across sites is housed at the MCRI, Melbourne, Australia. Data will only be published in de-identified, aggregated form.

#### Dissemination policy

Results will be published in peer reviewed publications.

## Discussion

This multicentre triple blind placebo controlled randomised trial will allow the definitive assessment of the efficacy of prednisolone in Bell’s palsy in children. It is likely that the findings will have a high impact on clinical management strategies beyond Australia and New Zealand.

### Current status

The study commenced in October 2015 and is recruiting. Recruitment and data analysis is expected to be completed in 2020.

## Abbreviations

AE: Adverse event
CEBU: Clinical Epidemiology And Biostatistics Unit
CI: Confidence Interval
CONSORT: Consolidated Standards of Reporting Trials
CHU-9D: Child Health Utility Index
CRF: Case Report Form
DSMB: Data Safety Monitoring Board
ED: Emergency Department
FPS-R: Faces Pain Scale Revised
HB: House Brackmann Scale
HREC: Human Research Ethics Committee
ITT: Intention-to-treat
MCRI: Murdoch Childrens Research Institute
NHMRC: National Health And Medical Research Council
PBS: Pharmaceutical Benefits Scheme (Australia)
PEDSQL: Pediatric Quality of Life Scale
PREDICT: Paediatric Research in Emergency Departments International Collaborative
RCH: Royal Children’s Hospital, Melbourne
RCT: Randomised Controlled Trial
REDCap: Research Electronic Data Capture
RR: Relative Risk
SAQ: Synkinesis Assessment Questionnaire
SD: Standard deviation
TSC: Trial Steering Committee
VAS: Visual Analogue Scale
